# MRI quantifies neuromuscular disease progression

**DOI:** 10.1016/S1474-4422(15)00320-8

**Published:** 2015-11-06

**Authors:** Sean C Forbes, Rebecca J Willcocks, William D Rooney, Glenn A Walter, Krista Vandenborne

**Affiliations:** Department of Physical Therapy (SCF, RJW, KV) and Department of Physiology (GAW), University of Florida, Gainesville, Fl, 32610, USA; Advanced Imaging Research Center, Oregon Health & Science University, Portland, OR, USA (WDR)

Several studies provide compelling support for the use of MRI as a sensitive non-invasive method to assess skeletal muscle disease progression in various neuromuscular diseases, including Duchenne muscular dystrophy^1,2^ and limb girdle muscular dystrophy type 2I.^3^ In *The Lancet Neurology*, Jasper Morrow and colleagues^4^ now report the sensitivity of MRI to track disease progression in 20 patients with Charcot-Marie-Tooth disease 1A and 20 patients with inclusion body myositis.

The investigators used a comprehensive study design that included magnetic resonance measures of muscle fat fraction, transverse relaxation time constant (T2), and magnetisation transfer ratio (MTR), along with relevant clinical functional tests (lower limb myometry, Medical Research Council score, Short-Form 36 Quality of Life Score, and Charcot-Marie-Tooth examination score or inclusion body myositis functional rating scale). In this study, the validity of the magnetic resonance measures was supported by strong correlations with clinical functional measures and the responsiveness to disease progression over 1 year was shown to be better with MRI than with the clinical functional tests. Notably, standardised response mean values were greater than 1 in inclusion body myositis and greater than 0·8 in Charcot-Marie-Tooth disease 1A, indicating that magnetic resonance measures are highly sensitive to disease progression and more responsive than established clinical measures. Even though Charcot-Marie-Tooth disease 1A progresses slowly, magnetic resonance measures detected substantial increases in disease pathological changes in 1 year. Thus, the encouraging results of Morrow and colleagues’ study might have a profound effect on clinical trials, potentially leading to a need for fewer participants to show efficacy or futility, shorter trials, and ultimately more rapid approval of treatments. An additional advantage of magnetic resonance measures compared with timed functional and strength measures, which are highly relevant to paediatric neuromuscular diseases, is that they are not dependent on participant motivation, an issue that has been of concern in Duchenne muscular dystrophy trials.^5^

Along with showing that MRI is a sensitive measure of disease progression in Charcot-Marie-Tooth disease 1A and inclusion body myositis, Morrow and colleagues also report that MRI-measured T2 and MTR are abnormal in these diseases even when fat fraction values are within normal limits. The authors interpret this to suggest that tissue water distribution changes before fat infiltration. Because MRI-measured T2 and MTR are affected by both fat and water content, the interpretation of these measures can be difficult in the context of neuromuscular diseases, and several analysis approaches are being developed to address this concern.^6–8^ Alternatively, magnetic resonance spectroscopy (MRS) could be used to more directly measure ^1^H_2_O T2 (figure) and MTR, and this would avoid the influence of fat.^9^ This method has already been applied to neuromuscular diseases^10,11 1^H_2_O T2 has been shown to differentiate boys with Duchenne muscular dystrophy and healthy individuals, even at a young age, when muscle fat fraction levels are normal.^10^ Furthermore, ^1^H_2_O T2 measured with MRS decreases with corticosteroid treatment in Duchenne muscular dystrophy, presumably because of reduced inflammation.^11^ Therefore, although lacking in spatial resolution, MRS is a high-fidelity approach to calculate ^1^H_2_O T2 and MTR measurements in neuromuscular diseases.

In view of the low prevalence of neuromuscular diseases, standardisation of methods over several sites will be crucial to the successful implementation of magnetic resonance biomarkers in clinical trials. Although challenging, when standardised protocols are carefully implemented across sites, several important magnetic resonance measures can be reproducibly obtained, including MRI-measured T2 and MRS measures of fat fraction and ^1^H_2_O T2.^12^ Similar to neuroimaging and musculoskeletal studies, for larger trials, incorporation of an infrastructure that enables automated or semi-automated processing, analysis, and quality control procedures that can detect and correct for system deviations, including instrument modifications and upgrades, will be particularly important.

Importantly, Morrow and colleagues showed the effectiveness of monitoring disease progression using magnetic resonance sequences commonly available on clinical scanners. Although the measures used were highly effective in tracking progression, future studies might include some developments. For example, the accuracy of fat fraction from the Dixon fat-water imaging sequence might be further improved by correcting for T2*, accounting for noise bias, and using a multipeak Dixon model specific to skeletal muscle.^13,14^

As suggested by Morrow and colleagues, the optimum outcome measures will depend on the pathophysiology, stage of disease, muscles affected, and intervention to be tested. This could include different types of magnetic resonance measures and analysis procedures. For example, targeting of specific muscles might be optimum for certain stages of a particular disease. Also, use of large regions of interest that encompass the entire muscle might be needed to detect pathological changes and improve responsiveness in some diseases, by contrast with small portions of the muscle, as used by Morrow and colleagues to measure T2 and MTR.

Overall, this study by Morrow and colleagues clearly shows the value of MRI to monitor disease progression and sets the stage for its potential use in clinical trials for Charcot-Marie-Tooth disease 1A and inclusion body myositis. With increased evidence of the validity and sensitivity of magnetic resonance biomarkers in neuromuscular diseases, the path for biomarker qualification (eg, their approval by the Food and Drug Administration) should be carefully explored, with the ultimate goal of using magnetic resonance measures as surrogate endpoints in clinical trials.

## Figures and Tables

**Figure:**
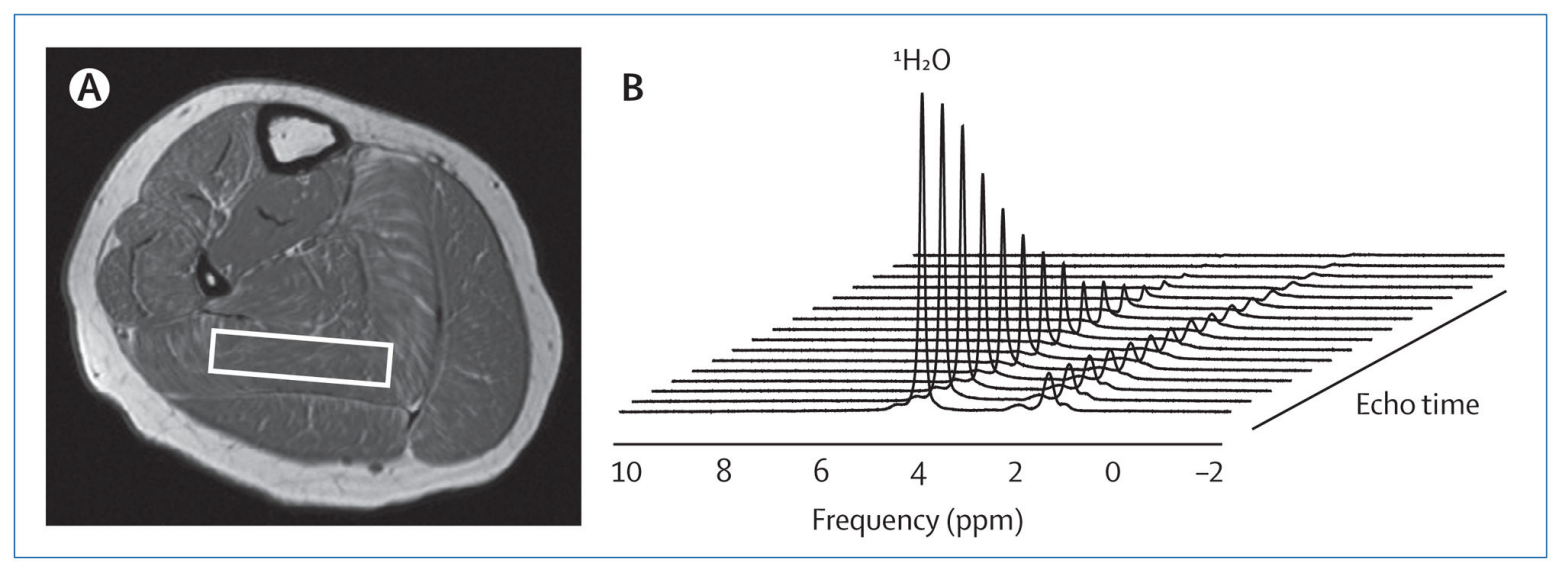
MRI and magnetic resonance spectroscopy of skeletal muscle in Duchenne muscular dystrophy (A) Magnetic resonance spin echo axial image of the lower leg of an 11-year-old boy with Duchenne muscular dystrophy. The white box indicates the position of voxel placement in which proton magnetic resonance spectroscopy data were obtained from the soleus muscle. (B) Single voxel spectroscopic relaxometry data were acquired using a non-linear increase in echo time (range 11–288 ms). Fitting the decay curve of the ^1^H_2_O peak to a mono-exponential model enabled a high confidence measure of ^1^H_2_O transverse relaxation time independent of lipid. In this example, ^1^H_2_O transverse relaxation time was calculated to be 36·5 ms in the boy with Duchenne muscular dystrophy, which is substantially longer than that of a typical unaffected boy of a similar age (27–29 ms).^9^ The proton spectra are displayed with water referenced at 4·7 parts per million (ppm) and the smaller resonances reported between 0·5 ppm and 2·8 ppm are from lipid protons. Images collected as part of the Imaging DMD study.

## References

[1] Willcocks RJ, Arpan IA, Forbes SC (2014). Longitudinal measurements of MRI-T2 in boys with Duchenne
muscular dystrophy: effects of age and disease progression. Neuromuscul Dis.

[2] Bonati U, Hafner P, Schädelin S (2015). Quantitative muscle MRI: a powerful surrogate outcome measure in
Duchenne muscular dystrophy. Neuromuscul Dis.

[3] Willis TA, Hollingsworth KG, Coombs A (2013). Quantitative muscle MRI as an assessment tool for monitoring
disease progression in LGMD2I: a multicentre longitudinal
study. PLoS One.

[4] Morrow JM, Sinclair CDJ, Fischmann A (2015). MRI biomarker assessment of neuromuscular disease progression: a
prospective observational cohort study. Lancet Neurol.

[5] Hoffman EP, Connor EM (2013). Orphan drug development in muscular dystrophy: update on two
large clinical trials of dystrophin rescue therapies. Discov Med.

[6] Azzabou N, Carlier P (2014). Fat quantification and T2 measurement. Pediatr Radiol.

[7] Rooney WD, Pollaro J, Forbes SC, Wang DJ, Vandenborne K, Walter GA (2011). Application of the extended phase graph technique to improve T2
quantitation across sites. Proc Intl Soc Mag Reson Med.

[8] Li K, Dortch RD, Welch EB (2014). Multi-parametric MRI characterization of healthy human thigh
muscles at 3. 0 T—relaxation, magnetization transfer, fat/water, and
diffusion tensor imaging. NMR Biomed.

[9] Machannab J, Schick F, Jacob S, Lutz O, Claussen CD (2000). An interleaved sampling strategy for MR spectroscopy in vivo:
applications on human calf musculature. Magn Reson Imaging.

[10] Forbes S, Willcocks R, Triplett W (2014). Magnetic resonance imaging and spectroscopy assessment of lower
extremity skeletal muscles in boys with duchenne muscular dystrophy: a
multicenter cross sectional study. PLoS One.

[11] Arpan I, Willcocks RJ, Forbes SC (2014). Examination of effects of corticosteroids on skeletal muscles of
boys with DMD using MRI and MRS. Neurology.

[12] Forbes SC, Walter GA, Rooney WD (2013). Skeletal muscles of ambulant children with duchenne muscular
dystrophy: validation of multicenter study of evaluation with MR imaging and
MR spectroscopy. Radiology.

[13] Triplett WT, Baligand C, Forbes SC (2014). Chemical shift-based MRI to measure fat fractions in dystrophic
skeletal muscle. Magn Reson Med.

[14] Loughran T, Higgins DM, McCallum M, Coombs A, Straub V, Hollingsworth KG (2015). Improving highly accelerated fat fraction measurements for
clinical trials in muscular dystrophy: origin and quantitative effect of
R2* Changes. Radiology.

